# A health app developer’s guide to law and policy: a multi-sector policy analysis

**DOI:** 10.1186/s12911-017-0535-0

**Published:** 2017-10-02

**Authors:** Lisa Parker, Tanya Karliychuk, Donna Gillies, Barbara Mintzes, Melissa Raven, Quinn Grundy

**Affiliations:** 1 Charles Perkins Centre, Faculty of Pharmacy, The University of Sydney, D17 6th floor, The Hub, Camperdown, Sydney, NSW 2006 Australia; 2 Australian Communications Consumer Action Network (ACCAN), Ultimo, NSW Australia; 3 Mental Health, Western Sydney Local Health District, North Parramatta, NSW Australia; 4 Critical and Ethical Mental Health Research Group, The University of Adelaide, Adelaide, SA Australia

**Keywords:** Mobile applications, Health policy, Health legislation, Professional ethics, Medical device legislation, Medical ethics, Privacy, Advertisements, Medical informatics

## Abstract

**Background:**

Apps targeted at health and wellbeing sit in a rapidly growing industry associated with widespread optimism about their potential to deliver accessible and cost-effective healthcare. App developers might not be aware of all the regulatory requirements and best practice principles are emergent. Health apps are regulated in order to minimise their potential for harm due to, for example, loss of personal health privacy, financial costs, and health harms from delayed or unnecessary diagnosis, monitoring and treatment. We aimed to produce a comprehensive guide to assist app developers in producing health apps that are legally compliant and in keeping with high professional standards of user protection.

**Methods:**

We conducted a case study analysis of the Australian and related international policy environment for mental health apps to identify relevant sectors, policy actors, and policy solutions.

**Results:**

We identified 29 policies produced by governments and non-government organisations that provide oversight of health apps. In consultation with stakeholders, we developed an interactive tool targeted at app developers, summarising key features of the policy environment and highlighting legislative, industry and professional standards around seven relevant domains: privacy, security, content, promotion and advertising, consumer finances, medical device efficacy and safety, and professional ethics. We annotated this developer guidance tool with information about: the relevance of each domain; existing legislative and non-legislative guidance; critiques of existing policy; recommendations for developers; and suggestions for other key stakeholders.

**Conclusions:**

We anticipate that mental health apps developed in accordance with this tool will be more likely to conform to regulatory requirements, protect consumer privacy, protect consumer finances, and deliver health benefit; and less likely to attract regulatory penalties, offend consumers and communities, mislead consumers, or deliver health harms. We encourage government, industry and consumer organisations to use and publicise the tool.

**Electronic supplementary material:**

The online version of this article (10.1186/s12911-017-0535-0) contains supplementary material, which is available to authorized users.

## Background

There is enormous optimism that mobile phone applications targeted at health and wellbeing (“health apps”) will improve access to cost-effective healthcare. The health apps industry is just a few years old, but already contains over 250,000+ products [1]. Governments and health professionals are actively involved in driving or funding app development [2, 3], endorsing existing apps [4, 5], and encouraging citizen use [6–8].

However, health app use could be harmful to consumers. For example, consumers may suffer loss of personal privacy, leading to reputational damage with subsequent financial, social, insurance or employment discrimination [9–11]. They may be misled into making unwanted purchases, or suffer direct financial losses [12]. Symptoms of ill-health may be exacerbated [13, 14], under-diagnosed [15], or over-diagnosed [16–18]. It is important for those involved in health app development (hereafter referred to by the umbrella term of “app developers”), regardless of whether they focus on the commercial, technical or health-related aspects, to be aware of the potential for negative outcomes, and to work towards producing apps that deliver benefits but also avoid, or at least minimise, possible harms.

To this end, government agencies that regulate health apps have begun to produce guidance to explain legislation and give advice on how to develop apps that are legally compliant and operate according to community values and expectations [19–22]. Industry groups have also released self-regulatory ethical codes that inform and instruct app developers on industry standards for professional practice (for example [23–25]). However, the full range of legal and professional guidance may not be readily obvious or accessible to app developers. This is partly because the pertinent guidance exists across a variety of sectors, including medical device, privacy, digital technology, advertising and finance, and is administered by separate and largely independent agencies [26]. App developers may not be aware of all relevant sectors, particularly those not specifically directed towards apps (for example, guidance about acceptable digital content more generally). App developers may also struggle with the depth of assumed knowledge in published guidance, particularly those from unfamiliar sectors. For example, medical device legislation is recognised as being confusing even to those who work professionally in medical device regulation [27, 28]. Further, similar terms can have differing meanings across sectors. For example, medical device legislation tends to use a narrow definition of ‘health services’, meaning the diagnosis, treatment and prevention of disease [29]. Privacy legislation tends to use the same term to mean assessing, maintaining or improving health [30].

App developers who do not conform to legal requirements may be penalised. Developers in the United States (US) recently settled with the Federal Trade Commission (FTC) for allegedly marketing health apps with unsubstantiated claims about their intended health function [31, 32]. There is a need for comprehensive and easily understandable guidance to assist app developers who wish to create legally compliant and ethically acceptable health apps. This paper describes the process of developing a guidance tool to assist app developers who are producing mental health apps distributed in the Australian market. The tool, the *App Developer’s Guide to Law and Policy* is publicly available through the Australian Communications Consumer Action Network (ACCAN) [33]. We expect that this tool could inform a template that would be transferrable to health apps more broadly, and could be used by developers working in other jurisdictions if populated with local laws and standards.

## Methods

This study was part of a larger project looking at the policy environment for mental health apps, which was funded by the Australian Communications Consumer Action Network (ACCAN), the peak body for consumer representation in the telecommunications industry. We use the term ‘mental health apps’ to include apps that offer, or claim to offer, one or more of: information, diagnosis, monitoring, treatment and support in relation to mental health symptoms, behaviours or illnesses. We chose to focus on mental health apps because digital mental health tools, including apps, are being heavily promoted by governments and the World Health Organization (WHO) [7, 34, 35] yet are associated with a risk of harm, including a heightened risk of stigma or discrimination associated with loss of privacy, and potential risks to consumer safety from under-treatment. Conversely, false positive diagnoses and over-diagnosis (for example true positive diagnoses of mild, self-limiting conditions where treatment carries no benefit) may be common, especially if the line between mental illness and normal variations in mood or in response to life events is not presented clearly.

The current study was informed by consumer advocacy and also by a policy analysis relevant to mental health apps. We used a broad definition of policy, encompassing the range of tools that provide regulatory oversight over mental health apps along their journey from development, distribution and selection for use by consumers. These policies included legislative guidance, industry self-regulation, post-market certification and evaluation programs, as well as general consumer tip sheets about selecting an app.

### Sampling

We used an iterative combination of broad and narrow search strategies to identify legal, industry and community guidance relevant to developers of mental health apps. We performed broad searches through PubMed and Google for literature on regulatory oversight of relevance to mental health apps. In order to capture legislative tools and key non-government influences, we searched for any policy authored by a well-known organisation that sought to influence the development, distribution or selection for use of health apps available in Australia and was applicable to mental health apps. Our inclusion and exclusion criteria are provided in Additional file 1.

We read through identified policies and hand searched their reference lists. We inductively identified six relevant regulatory sectors (privacy, security, digital content, promotion and advertising, consumer finance, and medical device) and searched purposively through the websites of the various Australian government departments providing oversight of each sector. Although we concentrated on Australian laws, we included legislation from other jurisdictions that was likely to be influential in Australia. For example, we included medical device legislation and guidance produced by the USA, the UK, the EU, and an international working group (the International Medical Device Regulators Forum, IMDRF), because the related Australian Government department (the Therapeutic Goods Administration, TGA) referred specifically to these policies. We also searched purposively through the websites of non-governmental organisations that had authored policies, looking for additional self-authored guidance, and following links and references.

We cross-checked our search strategy by circulating a preliminary list of identified policies to our research partners, including ACCAN. These partners confirmed that there were no major omissions from our list.

### Tool development

We read through all the policies to identify key elements of health apps that attracted regulatory oversight, and organised our developer tool around these domains. We drew further on policies and related references to provide information in each domain about legal requirements, compliance, and best practices. We defined best practice to mean non-legally-binding guidance contained within policies identified using the above process. We drew on the expertise of our research team, which included several mental health clinicians, academics with experience in commercial bias and health policy, and a telecommunications consumer group. We cross-checked the breadth of the tool by comparing it against various parameters of app quality listed in the published literature [36, 37].

## Results

We identified legislation and industry guidelines relevant to mental health apps from multiple, sector-specific domains. We identified 29 relevant policies, listed in Table 1. The key regulatory domains that we identified as being relevant to developers were:

1. consumer privacy
2. data security
3. content
4. promotion and advertising
5. consumer finances
6. medical device efficacy and safety
7. professional ethics

**Table 1:** List of identified policies relevant to developers of mental health apps in Australia

Regulatory domain/s addressed by policy	Policy Title	Jurisdiction	Author Sector
LEGISLATIVE GUIDANCE			
Consumer privacy, Data security	*Mobile Privacy: A Better Practice Guide for Mobile App Developers* [22]	Australia	Privacy
Consumer privacy, Data security	*Opinion 02/2013 on Apps on Smart Devices* [53]	EU	Privacy
Consumer privacy, Data security	*Mobile Health App Developers: FTC Best Practices* (and accompanying *Mobile Health Apps Interactive Tool*) [20, 21]	USA	Commerce
Consumer privacy, Data security, Promotion & Advertising	*Commission Staff Working Document on the Existing EU Legal Framework Applicable to Lifestyle and Wellbeing Apps* [6]	EU	Privacy, commerce, advertising
Consumer privacy, Data security, Promotion & advertising	*Marketing Your Mobile App: Get it Right from the Start* [68]	USA	Commerce
Consumer privacy, Data security, Content, Promotion & advertising, Consumer finances	*Mobile Apps: Emerging Issues in Media and Communications (Occasional paper 1)* [69]	Australia	Telecommunications
Promotion & advertising, Consumer finances	*App Purchases by Australian Consumers on Mobile and Handheld Devices: Inquiry Report* [12]	Australia	Commerce
Medical device efficacy & safety	*Regulation of Medical Software and Mobile Medical ‘Apps’* [29]	Australia	Health
Medical device efficacy & safety	*Software as a Medical Device (SaMD)* [70]	International	Health
Medical device efficacy & safety	*Mobile Medical Applications: Guidance for Industry and Food and Drug Administration Staff* [28]	USA	Health
Medical device efficacy & safety	*General Wellness: Policy for Low Risk Devices* [66]	USA	Health
Medical device efficacy & safety	*Guidance: Medical Device Stand-alone Software Including Apps (including IVDMDs)* [19]	UK	Health
INDUSTRY SELF-REGULATION			
Consumer privacy, Data security	*Mobile and Privacy: Privacy Design Guidelines for Mobile Application Development* [25]	International	Telecommunications
Consumer Privacy, Data security, Content, Promotion & advertising, Consumer finances	*App Store Review Guidelines* [23]	International	Digital media
Consumer Privacy, Data security, Content, Promotion & advertising, Consumer finances	*Developer Policy Center* [71]	USA	Digital media
Promotion & advertising	*Best Practice Guideline - Responsible Marketing Communications in the Digital Space* [64]	Australia	Advertising
Promotion & advertising	*Application of Self-Regulatory Principles to the Mobile Environment* [24]	USA	Advertising
POST-MARKET CONSUMER GUIDANCE - GOVERNMENT			
Consumer privacy, Data security	*Stay Smart Online: Mobile Devices* [72]	Australia	Privacy
Consumer privacy, Data security, Promotion & advertising, Consumer finances	*A Guide to Apps & In-app Purchases* [73]	Australia	Digital media
Consumer privacy, Data security, Content, Promotion & advertising, Consumer finances, Medical device efficacy & safety	*Safety and Quality Strategy in Mobile Health Apps* [5]	Spain	Health
Consumer privacy, Data security, Medical device efficacy & safety	*How to Choose a Health App* [74] and related references [75]	New Zealand	Health
Consumer finances, Medical device efficacy & safety	*Choose Wisely: Selecting Mobile Health Apps* [76]	USA	Defence
Medical device efficacy & safety	*VicHealth’s Top 10 Tips for Choosing a Healthy Living App; Guidelines for Creating Healthy Living Apps* [77] and related references [78–83]	Australia	Health
POST-MARKET CONSUMER GUIDANCE - INDSUTRY			
Consumer privacy	*Mobile App Advertising Guidelines: A Framework for Encouraging Innovation While Protecting User Privacy* [47] and related references [49]	USA	Advertising
Consumer privacy, Data security	*TRUSTed Apps Privacy Certification* [84]	USA	Privacy
Consumer privacy, Data security, Consumer finances, Medical device efficacy & safety	*myhealthapps.net* [85] and related references [86, 87]	UK	Health
Consumer privacy, Data security, Medical device efficacy & safety	*Doctor’s Guide to Choosing Health Apps that Really Work* [88] and related references [4]	USA	Health
POST-MARKET CONSUMER GUIDANCE - OTHER			
Consumer privacy, Data security, Promotion & advertising, Medical device efficacy & safety	*Mobile App Rating Scale (MARS)* [37]	Australia	Education
Medical device efficacy & safety	*PsyberGuide* [89]	USA	Health

Most policies addressed just one or two relevant domains (10/29 and 8/29 policies, respectively). We identified a dearth of comprehensive information collating the broad range of relevant legislative and ethical guidance in an easily accessible form that well-intentioned app developers could use to guide their work. We extracted and collated the information into a single guidance tool (see Additional file 2). The following sections provide domain-specific information on: why each domain is relevant to health apps, underlying regulatory principles, and dominant policies that provide regulatory oversight. We also discuss any regulatory shortcomings, and provide recommendations for app developers and other key stakeholders including consumers, app distributors, industries and governments. In each section, we include an excerpt from the guidance tool as an example of how these analyses have been translated for a health app developer audience in the Australian market [33].

### Consumer privacy

Health apps may access consumers’ identifying information and personal health information including diagnoses, symptoms and use of an app’s therapeutic services. Privacy legislation seeks to protect individuals by requiring consent for the collection, use, disclosure or retention of personal information including sensitive health information. It also requires that individuals be given access to, and have the option to correct, their information. In Australia, the applicability of privacy legislation to health apps depends on the type of developer, the developer’s annual revenue, and the nature of personal data collection. It is likely that most developers of health apps available in Australia will be bound by Federal privacy legislation (the *Privacy Act 1988 (Cth)*) [22, 38–40]. See Table 2 for key assessment questions to determine the applicability of privacy legislation in the Australian context.

**Table 2:** Sample developer guidance: Australian privacy laws and global best practices [33]

Does the app collect, use, disclose or hold any personal information?	
*Yes: Go to next question.*	
*No: You do not need to comply with any privacy legislation.*	
What kind of developer are you?	
• An individual or entity conducting a commercial activity	
o *Go to next question*	
• A federal public entity	
o *You must comply with the Australian Privacy Principles*	
• A State or Territory public sector entity	
o *You must comply with the Australian Privacy Principles*	
• An individual	
o *You are not required by law to comply with privacy legislation unless you are conducting commercial activity. However you should build privacy into your app’s design.*	
Does the app do, or claim to do, ANY of the following in ANY way?	
• Assess, maintain or improve a person’s physical or mental health, fitness or wellbeing?	
• Manage a person’s condition, disability or disease?	
• Diagnose or treat a person’s illness or disability, or injury?	
• Record a person’s health information?	
*Yes: you must comply with the Australian Privacy Principles: This means that you must:*	
• *Manage personal information in an open and transparent way (this includes having a clearly expressed Privacy Policy)*	
• *Adhere to principles about how personal information can be collected, used and shared*	
• *Keep personal information secure*	
• *Ensure people can access and correct their personal information*	
*No: you are not required by law to comply with privacy legislation. However, you should aim for privacy by design.*	

Current policies aimed at protecting the privacy of mental health app consumers are insufficiently enforced: many apps do not provide privacy policies, and those that do often deliver information via impenetrable documents that are difficult for non-technical users to understand [41–44]. In addition, some practices are unacceptable and/or unlawful even with full disclosure and consent [45]. For example, collection and sharing of sensitive consumer information, including mental health information, for reasons unrelated to the main function of the app is not allowable under Australian legislation [22]. This is because knowledge of sensitive information can have significant repercussions for individuals, including employment, insurance, and financial discrimination [45, 46]. The complexity of the so-called “mobile eco-system” is so high that app developers and app users, may not know who data is shared with, and for what purpose [22, 47]. Privacy policies rely heavily on consumer complaints, but consumers may not know the law and may be unaware of sharing of personal data or how the data is being used to define their reputation in terms of employability, insurability or fiscal dependability.

#### Recommendations

In the tool (Additional file 2), we recommend that app developers ensure that any practices to share/sell consumer data are transparent and appropriate with regard to protection of privacy and the interests of the general public. This is in keeping with other practices that are required to respect individual interests, such as human research, where formal codes of ethics mandate the consideration of issues such as transparency, consent, and overall acceptability of research practices [48]. At the moment, it is likely that developers of apps available in Australia will continue to share/sell the personal or health data of their consumers with third parties without specific consent, since this is an important business strategy for many apps [49] and it may be allowable in other jurisdictions [50–52]. In addition, enforcement of legal restrictions may be difficult. For example, it may be impossible for legislators to follow the chain of entities who have access to the data, and difficult for them to enforce local laws in relation to products that originate from other jurisdictions [40].

As a start, we encourage public discussion and advocacy in this field with the aim of increasing public awareness of current practices and possible implications, and generating public input into regulatory standards or a government-endorsed code about what is and what is not acceptable in terms of health app data-sharing. Consumers and governments should also exert pressure on industry bodies to adopt higher standards that improve consumer privacy. For example, app distributors should enforce the inclusion of user-friendly privacy policies for all health apps, device manufacturers should ensure that privacy-friendly settings are the default setting on phones, and data-brokers and other third parties should develop strong self-regulatory systems around practices for obtaining, sharing, using and retaining consumer data.

### Data security

While privacy legislation requires certain security measures, there is no specific security legislation that protects consumer data from attack by third parties. See Table 3 for an excerpt from the guidance tool. Many health apps have inadequate security arrangements. For example, an external study of apps recommended on the English National Health Service (NHS) Health Apps Library found serious security inadequacies in many apps, including lack of encryption for stored data in 93% of apps (73/79) and transmission of unencrypted identifying information in 29% (23/79) [43].

**Table 3:** Sample developer guidance: Australian security laws and global best practices [33]

If your app is subject to the Privacy Act 1988, then you must take reasonable steps to protect the personal information you collect, store or share. Even if your app is exempt from the *Privacy Act 1988*, you should ensure the app is secure.	
There is no specific security law that app developers must follow. Instead, developers should use a risk-based approach to decide on the most appropriate level of security. The more sensitive the personal information collected, the stronger your security should be. Health information is highly sensitive, so apps that collect, store or share health information should adopt the strongest security measures.	

#### Recommendations

In our tool (Additional file 2), we recommend that developers pay strict attention to the security advice contained within legislative guidelines, and implement strong security protections on all health apps. There are a number of government and industry documents that provide useful detail on security measures for health app developers [22, 53, 54].

More broadly, we encourage greater public debate and discussion to increase public awareness about the inherent insecurities of digital technology. We encourage industry to prioritise innovation in health app security.

### Digital content

Apps and app promotional material that contain easily accessible but inappropriate content may cause serious offence to or be unsuitable for viewing by consumers (including children) and communities. Apps available in Australia are bound by Federal Government legislation that regulates online content (the Online Content Scheme under Schedules 5 and 7 of the *Broadcasting Services Act 1992*) [55]. See Table 4 for sample guidance. Many are also subject to the content policies of commercial app stores.

**Table 4:** Sample developer guidance: Australian digital content laws [33]

Does your app contain ANY of the following?	
• Images of child sexual abuse or instructions in paedophilia	
• Depictions of gratuitous or exploitative violence including sexual violence	
• Depictions of actual or exploitative sexual practices including bestiality or incest	
• Detailed instruction or promotion of crime or violence including the use of illicit drugs or terrorist acts	
*Yes: your app is prohibited by law and must not be available for download in Australia. Distribution, promotion or possession of this kind of app is a criminal offence. Offensive content in your promotional materials is also prohibited.*	
*No: Check your social media channels regularly – if offensive material is posted by users in relation to your app, you must remove this promptly*.	
Your app must have an appropriate age-classification. Check your app for mature themes and language, violence, sex, drug use and nudity.	

Government legislation on digital content may not be effective because easily accessible information about the role and remit of the legislation with regard to apps is limited [55–57]. For example, the Australian legislation does not specifically mention apps, referring only to films and games. Furthermore, products are only regulated under this legislation if they are “provided on a commercial basis (i.e. for a fee)” [55] yet many commercially developed apps are monetised without an up-front consumer fee (e.g. via advertising revenue or sale of consumer data.) The legislation may also be ineffective because it relies on consumers to identify and file a complaint about offensive material.

In contrast, content policies from the major app stores of Apple and Google Play are likely to be very effective indeed, their dominant influence on the app market enabling them to act as the de facto regulators of acceptable content standards. This situation is problematic, since Apple and Google Play do not provide detailed information about their standards. Apple, for example, gives the following vague guidance:

We will reject apps for any content or behavior that we believe is over the line. What line, you ask? Well, as a Supreme Court Justice once said, 'I'll know it when I see it.' And we think that you will also know it when you cross it [23].

The values underpinning these kinds of guidelines are opaque to the community and therefore difficult to critique or challenge. This opens the door to both unreasonable and unnecessary censorship and offensive communication: potentially depriving the public of certain material likely to be considered acceptable within the general community, and enabling unbridled release and distribution of offensive or inappropriate content (for example, forums for mental health app users might be used as a means to engage in cyber-bullying or to exchange advice on self-harm).

#### Recommendations

In our tool (Additional file 2), we recommend that app developers be aware of and adhere to legislation around digital content. Due to the near market-monopoly positions of Apple App Store and Google Play Store, legislation will likely play less of a role than the content guidelines from these major app distributors. Consequently, we also urge the public and governments to exert pressure on app stores to be transparent about their standards for content, thus enabling critique of their practices and facilitating change where needed.

### Promotion and advertising

Deceptive or misleading promotion of health apps is inconsistent with consumer rights in trade and commerce, may compromise user health and safety, and could contribute to excessive societal health costs by encouraging indiscriminate or inappropriate use of related health services. That is, apps may give false expectations of benefit, or may engender unrealistic expectations about wellbeing and unnecessarily medicalise the ups and downs of daily living [16, 58].

Promotional material includes the app descriptor in a commercial app store or developer website, and any information provided about the app under the auspices of the developer, such as external endorsements reproduced within an app descriptor, and information or reviews hosted on an app developer’s social media channels, even if that information is posted by a third party. Several regulatory policies pertain to advertising materials. First, consumer marketing legislation seeks to protect consumers from deception or misleading claims about products or services that they obtain [59]. Second, legislation about advertising of regulated health services has tighter restrictions on advertising techniques (for example, banning the reproduction of a user review or personal testimonial within an advertisement for health services) [60]. Third, medical device legislation, may be applicable to some health apps, and prohibits: advertising which is not for the device’s intended purpose; making reference to a serious disease without prior regulatory approval; or health professional endorsement [61]. Finally, the advertising industry has its own voluntary codes that set industry standards for various aspects of advertising, including appropriateness of content [62, 63] and acceptable advertising practices [64]. See Table 5 for sample guidance.

**Table 5:** Sample developer guidance: Australian advertising laws and global best practices [33]

Do the app’s promotional materials accurately reflect what the app provides?	
*Promotional materials that are deceptive or misleading are prohibited under the Australian Consumer Law.*	
Is the promotional material likely to be seen by an audience that includes children?	
*Advertising industry standards recommend that promotional material should not be offensive and should be age-appropriate*	
Are there any up-front or in-app charges associated with downloading or using the app?	
*The Australian Consumer Law states that you must be clear and transparent in your advertising about the cost of the app, including whether in-app purchases are required for full functionality.*	
Does downloading or usage of the app require extraordinary amounts of data?	
*You should provide information upfront about data usage*.	

Although this regulatory oversight appears extensive, its functionality is limited by lack of funds and a heavy reliance on consumer-initiated complaints. The consumer complaints mechanism may be inadequate if consumers are unsure or unaware about the extent of consumer protection laws (e.g. applicability to apps that were free to download and/or developed outside Australia) or where to make complaints, and if government agencies have inadequate resources available to respond to most concerns. In addition, the existing oversight does not provide sufficient protection for vulnerable consumers. It seems likely that many users of mental health apps will be distressed or worried and could be more susceptible to predatory advertising about unrelated topics that are a common source of anxiety (for example, weight, body image, sexual prowess). However, there are no restrictions on the content of advertisements that are targeted at mental health or other vulnerable app users in, for example, banner ads viewed while accessing or using the app, or in personalised ads that pop up during unrelated internet browsing. Further compounding the difficulty in regulating this kind of content is the fact that these types of advertisements are generally not within the developer’s control, but the product of ad libraries that developers choose to embed in their app.

#### Recommendations

We recommend that app developers become aware of and adhere to legislative and industry standards on advertising (see Additional file 2). For health apps, this includes NOT making claims that are unsupported by clinical evidence. In order to increase the levels of consumer protection, we encourage increased consumer awareness of consumer complaints mechanisms, together with increased funding of government consumer protection agencies. Consumer-focused legislation pertaining to trade promotion and advertising has proven to be a powerful regulator of health apps in other jurisdictions and may be the most suitable tool to provide strong oversight in this field [31, 32]. In addition, legislation around promotion and advertising may be particularly suitable as a primary regulator for health apps because apps are consumer-targeted and because it is applicable to all kinds of apps, not just those that fulfil the specialised definition of a medical device. We also urge the public and governments to place pressure on third parties to self-regulate: creating high standards for the entire chain of actors who participate in behavioural advertising in order to prevent inappropriate practices that target vulnerable consumers.

### Consumer finances

Most app stores adhere to industry codes of practice around electronic transactions [65] and will provide a refund if an app does not function as described. We recommend in the tool that app developers who sell their products directly to consumers should follow similar business practices. Other financial matters are less regulated. App subscriptions can be unending, requiring active cessation by consumers, and there are no government recommendations about limiting the promotion of in-app purchases, except for apps directed at children [12]. See Table 6 for sample guidance.

**Table 6:** Sample developer guidance: Australian consumer finance laws and global best practices [33]

Does your app contain in-app purchases?	
*You should provide information about in-app purchases in promotional materials and in the app. The Australian Consumer Law requires you to indicate whether in-app purchases are required for full functionality. If your app is targeted at potentially vulnerable users such as children or people living with mental illness, you should not repeatedly offer users in-app purchases.*	
Do you sell your app directly to consumers (e.g. via your own website)?	
*You should have an obvious and accessible process for refunding consumers the costs of downloading or using the app if it fails to meet consumer guarantees.*	

#### Recommendations

We recommend that app developers conform to acceptable refund practices and avoid making repeated offers for in-app purchases, particularly within the context of a mental health app (Additional file 2). We have recommended that app developers adhere to consumer advertising legislation, and this includes being transparent in advertising materials about the financial costs associated with app download and use.

In addition, we recommend that consumers and governments exert pressure on app distributors to exclude apps that promote in-app purchases within apps targeted at vulnerable consumers. We also urge distributors to mandate time-limits on subscription payments when apps remain unused for long periods.

### Medical device efficacy and safety

Health apps that are intended to function as aids or alternatives to traditional medical services (for example by offering diagnosis, prevention, monitoring or treatment) carry particular risk of harm to users’ health and safety. These kinds of health apps may harm user health, by, for example, aggravating symptoms, delaying diagnosis, or driving unnecessary diagnosis and treatment [13–15, 18]. Consequently, these apps fall under medical device legislation, and may be subject to a high level of government scrutiny and control. There are multiple demands on medical device manufacturers, such as ensuring that the device is recorded on a central register, keeping records of quality control practices, collecting clinical evidence about the outcomes of device use and documenting any adverse events. The legislative requirements vary depending on the type of medical device and its associated risk of harm [27].

The application of medical device law to health apps is in a state of flux: the health apps industry is new and there has been debate globally over which health apps, if any, would be classed as medical devices. Recent publications by medical device regulators in several jurisdictions have sought to alleviate this confusion by releasing guidelines specifically about apps for health and/or wellbeing [6, 19, 29]. The Australian guidelines, for example, indicate that health apps will come under medical device legislation if they fulfil the definition of a medical device. The Food and Drug Administration, the US government agency responsible for medical device regulation, has also advised that it will not pursue regulatory oversight of health apps that fit their definition for low risk of harm, regardless of whether or not they fill the legal definition of their medical device law [66]. It is not yet clear whether other jurisdictions will officially adopt a similar policy. See Table 7 for sample developer guidance.

**Table 7:** Sample developer guidance: Australian medical device laws and global best practices [33]

Is the focus of the app ANY of the following?	
• A specific disease, injury or disability? [This DOES include medical diagnoses and conditions (e.g. depression, eating disorder). It does NOT include symptoms or conditions that are not classified as a medical disease (e.g. stress, trouble concentrating, difficulty sleeping).]	
• An anatomical or physiological *process*? [This DOES include things like the sleep cycle. It does NOT include general well-being.]	
• Control of conception	
*Yes: go to next question. No: it is unlikely that your app is a medical device.*	
Does the app claim that the output from the device can prevent or treat a specific disease, injury or disability or directly influence an anatomical or physiological process? Answer no if the app ONLY provides tips and advice on prevention or treatment.	
*Yes: go to next question. No: it is unlikely that your app is a medical device.*	
Does the app collect user-generated data*?	
*Yes: go to next question. No: it is unlikely that your app is a medical device.*	
Does the app deliver individualised health messages on the basis of user-generated data?	
*Yes: it is likely that your app is medical device; go to next question to assess its likely risk-categorisation and regulatory requirements.*	
*No: it is unlikely that your app is a medical device.*	
Does the app allow direct diagnosis or monitor a vital physiological process?	
*Yes: Your app is likely a Class IIa (low-medium risk), Class IIb (medium-high risk) or Class III (high risk) medical device. All of these apps must be assessed by the Australian Therapeutic Goods Administration or must hold an equivalent certificate from a European Notified Body.*	
*No: Your app is likely a Class I (low risk) medical device.You must conform to the Australian Therapeutic Goods Administration’s Essential Principles for safety and performance but unless your app has a direct measuring function (e.g. wearables) your app does not require external assessment by the Therapeutic Goods Administration . You must be able to provide evidence of conformity to the Therapeutic Goods Administration upon request.*	
*USER GENERATED DATA is any information entering the app that comes from the user. Apps may rely on user-generated data to generate tailored messages to users via algorithms, calculators, coaches or other means. If an app delivers tailored health messages, it may be classified as a medical device. Example health messages include:	
• Diagnosis: e.g. The user has…	
• Prognosis: e.g. The user is at risk of …	
• Monitoring: e.g. The user’s disease is getting better / worse, or is stable / unstable	
• Advisory: e.g. The user should pursue a particular behaviour or use a product or service in a particular way (eg specifying dose or timing)	
Tips: An app is unlikely to be classified as a medical device, if the app only ever:	
• Indicates the risk that a population group has of developing a disease	
• Provides general advice about a “healthy lifestyle” (such as limiting smoking and alcohol use, getting sufficient exercise);	
• Provides links to support groups	
• Gives generic advice to “seek help”	
• Provides education about disease, anatomy or physiology	
• Reminds users to take medications	
• Monitors general health, fitness, wellbeing or the menstrual cycle (except if it investigates a specific physiological process)	
• Stores user-generated data for later review by a health professional	

We have identified several potential problems with policy oversight in the medical device domain. Medical device legislation is complicated and it may be very difficult to understand whether a particular health app comes under the legal definition of a medical device [27, 28]. The matter turns on interpretations of specialised concepts such as what constitutes a disease, and definitions of diagnosis, monitoring or treatment. In addition, for any given medical device, legislative instruments tend to rely on a risk-based classification to determine the particular suite of legislative demands, but developers may be ill-equipped to identify levels of risk or potential harms. Health app developers may be in breach of medical device law if they do not recognise when medical device legislation is relevant, do not think to check – or do not understand – their legal requirements. There are several reasons why the law may not sufficiently protect the health of consumers and communities: even if apps are included on medical device registers, apps change and update frequently and registers may not be designed to deal with this; medical device legislation is at least partly reliant on consumer complaints, particularly for low risk items, but consumers may not know to use this sector for complaint; and medical device law does not cover societal harms that may arise from predatory overdiagnosis [18]. In sum, it may be easy for developers and app stores with benign or malign intentions to produce and distribute health apps that do not conform to medical device law or that risk delivering health harms to consumers and society.

#### Recommendations

We recommend that all health app developers familiarise themselves with the legal requirements of their local medical device regulator, particularly in relation to any requirements for supporting evidence of efficacy (see Additional file 2). However, we also acknowledge that most health apps will not fit the legal definition of a medical device. Regardless, we encourage all developers of health apps to provide information to users about factors that may influence the apps’ likely efficacy and risk of harms (for example, whether or not the app was developed in conjunction with health experts, whether or not it incorporates recognised healthcare guidelines or is informed by other scientific evidence, and who is funding the app). Particularly, transparency about the scientific evidence (or lack of evidence) underpinning the app’s effectiveness on health outcomes is key.

Medical device legislation seems ill-suited to regulate health apps; the laws are complex, burdensome, and seemingly ineffective at regulating health and safety in a rapidly changing field. It may be more appropriate for a dedicated government body to take oversight, providing a central repository for consumer complaints and registration of adverse outcomes in all domains (e.g. loss of privacy, financial embarrassment, misleading claims, health harms). Such a body might exist as a division of an existing government entity that manages other electronic or digital health matters, or that provides consumer trade protection, and could liaise, as necessary with medical device regulators and other government departments. This body should be charged with considering the impact of health apps upon both individual consumers and more broadly upon society, and should act to regulate in a manner that protects consumer health and safety, and protects the public interest in avoiding “too much medicine.”

### Professional ethics

Governments and industry bodies advise health app developers on additional parameters of app quality that are not covered in legislation, including accountability, truthfulness and transparency [67]. See Table 8 for sample guidance.

**Table 8:** Sample developer guidance: professional practice [33]

There are standards of professionalism that set some health apps apart. Here’s a checklist to see if your app can compete:	
I have identified myself as the developer and provided contact information in the app, in store and on promotional materials.	
I have identified the authors of the app content by: disclosing authorship; providing author credentials; citing all sources; attributing all intellectual property	
I have disclosed all funding sources for the app, including commercial partners, in the promotional materials and in the app itself	
I have disclosed my business model so consumers understand how they are paying for the service.	
I have provided scientific evidence to support the claims about what the app can do.	
If I’m making a health claim, I have provided clinical evidence	
I have provided an easily accessible and understandable privacy policy	
I have obtained consumers’ fully informed consent	
I have carefully selected third party partners so that I only work with partners that are transparent and accountable about how they collect, store and share user data.	
I have designed my apps to be usable by all consumers including people with specific user needs such as those people with vision, hearing or dexterity impairments.	

### Recommendations

We recommend that app developers should provide details on key stakeholders, scientific sources, and the app’s monetisation strategy. Developers should ensure that their privacy policies, privacy practices and consent processes are not just box-ticking exercises that aim to limit their own legal liability, but actually enable consumers to protect their privacy while using apps (See Additional file 2).

## Discussion

We have identified legislation and industry guidelines relevant to mental health apps from multiple, sector-specific domains. We extracted and collated the information into a single tool with the aim of providing guidance to app developers in this field. We anticipate that this tool will be useful for informing mental health app developers about legal requirements, industry standards, and professional expectations. Ultimately, we hope that this will lead to mental health apps that deliver better outcomes for app consumers and the public, and mitigate the risk of breach for app developers. We anticipate that mental health apps developed in accordance with this tool will be more likely to: conform to regulatory requirements, protect consumer privacy, protect consumer finances, and deliver health benefit; and less likely to: attract regulatory penalties, offend consumers and community groups, mislead consumers, and deliver health harms.

The process and result of preparing this developer tool highlighted several important problems with regulation of mental health apps. Legislation is siloed in separate government departments. We note above that many laws depend on self-declared compliance and consumer complaints, and that some laws are poorly expressed to facilitate this. However, the fragmented nature of the regulatory oversight adds an additional problem. Consumers who are motivated to complain may not know where or how to direct their concerns. For example, consumers who experience worsening symptoms associated with the use of an app to manage stress may wonder whether complaints and concerns should be directed at consumer protection agencies, medical device agencies or app stores. This means that illegal and potentially harmful apps may persist in the public domain. We suggest that a more cohesive regulatory framework might assist in providing effective oversight of health apps. This might include, for example, having a single point of consumer access for complaints about any aspect of health apps, and a central register for adverse health outcomes from app use, regardless of their assigned level of risk-of-harm. There is ample scope for further research in this area.

### Limitations

We prepared this tool with reference to Australian legislation and international influences. Although it will be of most immediate practical use to app developers making products for the Australian market, it can be adapted to suit other jurisdictions by inserting local laws and guidelines into the existing template. There is, however, a particular lack of research on health app content and policy in countries outside of the Western, English speaking nations, and we would advocate for further work here [36]. The tool is not intended to provide legal advice, but it has been prepared by a team with expertise in relevant sectors, and we think that it provides valuable guidance to the range of laws and industry best practices that pertain to health apps. The tool may have additional limitations associated with the sampling methods. That is, we may have missed relevant sectors or important documents, although our inductive strategy for identifying policies was designed to minimise this. The tool will, inevitably, become out of date as laws and guidelines change, but can be readily updated with revised policies.

## Conclusion

There has been much written about the potential for health apps to deliver benefits but, as with many health innovations, less attention has been paid to considering, measuring or minimising the possible harms.

We have created a tool that is suitable for developers who wish to create legally compliant mental health apps that accord with high professional standards of consumer care and protection. We anticipate that it will be helpful with navigating the complex array of legislative guidelines and industry best-practice standards across multiple relevant domains. The tool can be used at all levels of app production, from inspiration through to development and evaluation, and may also be a suitable template for post-market assessment. We encourage government, industry and consumer organisations to use and publicise the tool.

Other key stakeholders in the health apps industry, including consumers, governments, app distributors and third parties, also have a role to play in improving consumer protection and we hope that our suggestions for action might stimulate further discussion.

## Additional files

Additional file 1: Inclusion and exclusion criteria for our policy sample. Inclusion and exclusion criteria for our policy sample. (DOCX 58 kb)

Additional file 2: App developer’s guide to law and policy. A guidance document to assist app developers create health apps that are legally compliant and in line with industry and community standards. (PDF 711 kb)

## Abbreviations

ACCAN: Australian Communications Consumer Action Network
Cth: Commonwealth
EU: European Union
FTC: Federal Trade Commission
Health Apps: Mobile phone applications targeted at health and wellbeing
IMDRF: International Medical Device Regulators Forum
IVDMD: In vitro diagnostic medical devices
NHS: National Health Service
TGA: Therapeutic Goods Administration
UK: United Kingdom
USA: United States of America
WHO: World Health Organization
