# Supplementary material for: A health app developer’s guide to law and policy: a multi-sector policy analysis
Source: BMC Med Inform Decis Mak. 2017 Oct 2;17:141. doi: 10.1186/s12911-017-0535-0 (PMC5625720; doi:10.1186/s12911-017-0535-0)
Supplement: Supplementary file 1 — Inclusion and exclusion criteria for our policy sample. Inclusion and exclusion criteria for our policy sample. (DOCX 58 kb) [file 12911_2017_535_MOESM1_ESM.docx]

**Additional File 1: Inclusion and exclusion criteria for our policy sample**

Inclusion criteria:

- Providing guidance with one or more of: a legal basis, an enforcement mechanism, clear evidence of implementation
- Pertaining to apps on a mobile platform
- Applicable to apps that provide information, diagnosis, monitoring, treatment, or support related to mental health
- Published by an active, prominent entity defined as: government; major university, or healthcare institution; multinational corporation; or national organisation including peak industry, consumer and non-profit bodies
- Published in the last ten years
- An Australian focus or a clear influence on the Australian context

Exclusion criteria:

- Not freely available in English
