# Supplementary material for: A health app developer’s guide to law and policy: a multi-sector policy analysis
Source: BMC Med Inform Decis Mak. 2017 Oct 2;17:141. doi: 10.1186/s12911-017-0535-0 (PMC5625720; doi:10.1186/s12911-017-0535-0)
Supplement: Supplementary file 2 — App developer’s guide to law and policy. A guidance document to assist app developers create health apps that are legally compliant and in line with industry and community standards. (PDF 711 kb) [file 12911_2017_535_MOESM2_ESM.pdf]

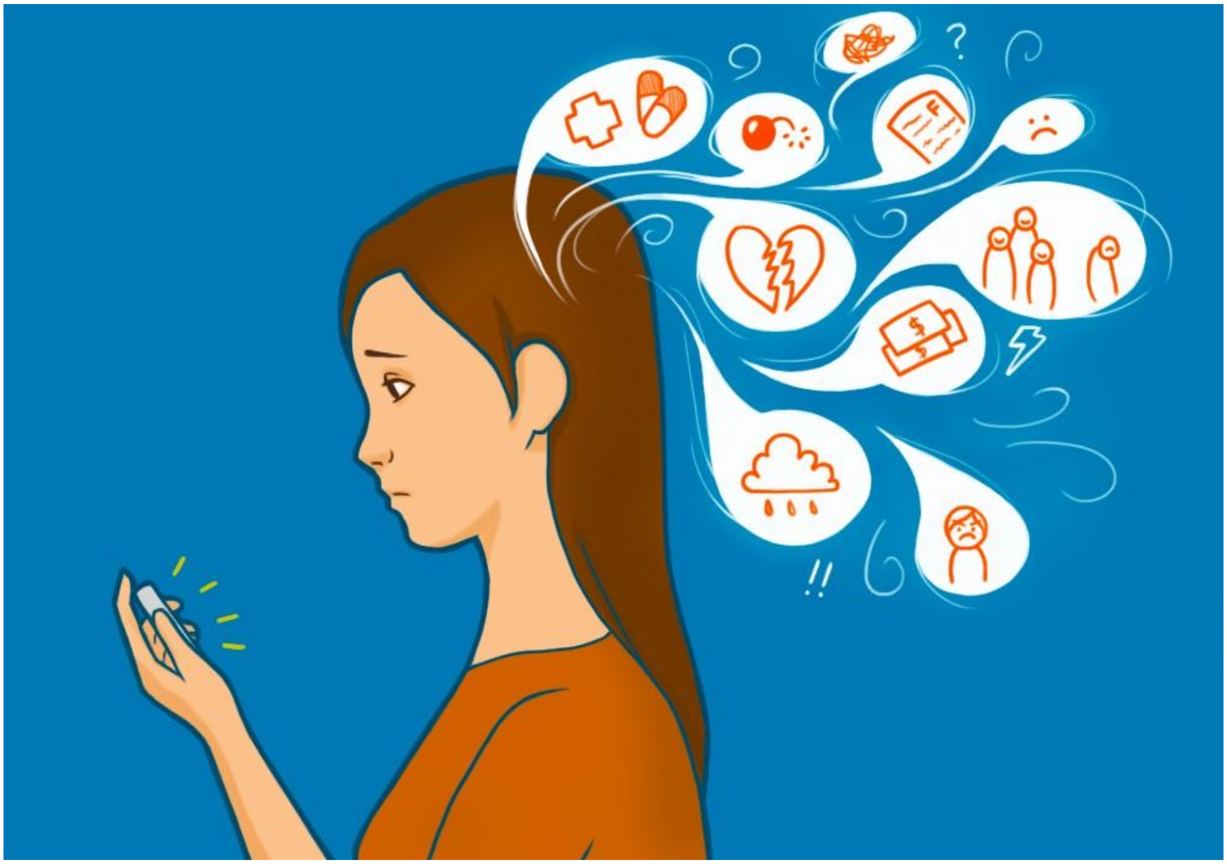

# App developer's guide to law and policy:

## Creating quality mental health apps

Produced in cooperation with The University of Sydney and the Australian Communications Consumer Action Network (ACCAN).

THE UNIVERSITY OF SYDNEY

accan

Copyright © The University of Sydney, 2017

# Developing a mental health or wellbeing app?

Find out which laws and standards apply to you. This tool:

- Helps you identify which Australian laws apply to your app
- Suggests best practices to promote consumer confidence in your app

While this is not legal advice, it will point you in the direction of resources to help ensure your health app is legally compliant and in line with industry and community standards.

This tool will cover six areas of the law: privacy, security, content, advertising, financial, and medical device. But it also provides a checklist to ensure your app meets the highest standards of professionalism.

Copyright © The University of Sydney, 2017

# Privacy

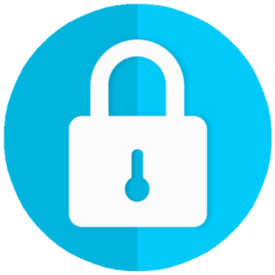

Health information is one of the most sensitive types of personal information. For this reason, the law requires extra protections when handling health information. Most health apps are covered by the Privacy Principles of the Privacy Act https://www.oaic.gov.au/privacy-law/privacy-act/australian-privacy-principles. Answer these questions to find out how it applies to you:

**1.1 Does the app collect, use, disclose or hold any personal information?**

**YES** Go to Question 1.2

**NO** You do not need to comply with any privacy legislation.

**1.2 What kind of developer are you?**

| | |
|---|---|
| **An individual or entity conducting a commercial activity** | Go to Question 1.3. |
| **A federal public entity** | You **must** comply with the Australian Privacy Principles https://www.oaic.gov.au/privacy-law/privacy-act/australian-privacy-principles |
| **A State or Territory public sector entity** | Your app is covered by your State or Territory's privacy legislation and you **must** comply with their Privacy Principles https://www.oaic.gov.au/privacy-law/other-privacy-jurisdictions |
| **An individual** | You are not required by law to comply with privacy legislation unless you are conducting commercial activity. However, you **should** build privacy into your app's design. Here's how: https://www.oaic.gov.au/agencies-and-organisations/guides/guide-for-mobile-app-developers |

**1.3 Has your business had an annual turnover of more than $3,000,000 in any financial year since 2002?**

**YES** You **must** comply with the Australian Privacy Principles https://www.oaic.gov.au/privacy-law/privacy-act/australian-privacy-principles

**NO** Go to Question 1.4.

Copyright © The University of Sydney, 2017

**1.4 Does the app do, or claim to do, ANY of the following in ANY way?**

- Assess, maintain or improve a person's physical or mental health, fitness or wellbeing?
- Manage a person's condition, disability or disease?
- Diagnose or treat a person's illness or disability, or injury?
- Record a person's health information?

**YES** You **must** comply with the Australian Privacy Principles https://www.oaic.gov.au/privacy-law/privacy-act/australian-privacy-principles

**NO** You are not required by law to comply with privacy legislation. However, you **should** aim for privacy by design. Here's how: https://www.oaic.gov.au/agencies-and-organisations/guides/guide-for-mobile-app-developers

## PRIVACY BOX

The Australian Privacy Principles outline how to collect, use and manage personal information. You **must**:

- Manage personal information in an open and transparent way (this includes having a clearly expressed Privacy Policy)
- Adhere to principles about how personal information can be collected, used and shared
- Take measures to maintain the quality of personal information Keep personal information secure
- Ensure people can access and correct their personal information

Here's how: https://www.oaic.gov.au/agencies-and-organisations/guides/guide-for-mobile-app-developers For more information, see the website of the Office of the Australian Information Commissioner (OAIC): https://www.oaic.gov.au/privacy-law/privacy-act/australian-privacy-principles

Copyright © The University of Sydney, 2017

# Security

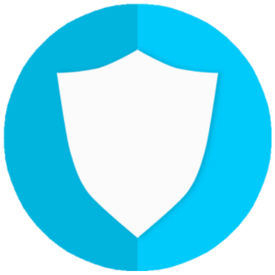

If your app is subject to the Privacy Act, then you must take reasonable steps to protect the personal information you collect, store or share. Even if your app is exempt from the Privacy Act, you **should** ensure the app is secure.

There is no specific security law that app developers must follow. Instead, developers **should** use a risk-based approach to decide on the most appropriate level of security. The more sensitive the personal information collected, the stronger your security should be. Health information is highly sensitive, so apps that collect, store or share health information **should** adopt the strongest security measures.

## WHY SECURITY MATTERS

The FTC recently found that 12 popular health apps transmitted personal data http://adage.com/article/privacy-and-regulation/ftc-signals-focus-health-fitness-data-privacy/293080/ including names, email addresses and unique device IDs to 76 third parties. Some third parties received personal data from more than one app, allowing them to put together a more complete picture of the user. This could greatly affect a consumer's insurance premiums, for example, if this data is then sold.

Here's how you can ensure your app is secure:

- OWASP Mobile Security Project - Top Ten Mobile Controls: https://www.owasp.org/index.php/Projects/OWASP_Mobile_Security_Project_-_Top_Ten_Mobile_Controls
- Association for Data-driven Marketing & Advertising (ADMA) Code of Practice: https://www.adma.com.au/compliance/code-of-practice
- Office of the Australian Information Commissioner (OAIC) Guide to Securing Personal Information: https://www.oaic.gov.au/agencies-and-organisations/guides/guide-to-securing-personal-information

Copyright © The University of Sydney, 2017

# Content

The law prohibits offensive online material, including offensive content in smartphone apps. In particular, your app **must not** expose children to offensive or unsuitable material. See if these rules apply to your app:

**3.1 Does your app contain ANY of the following?**

- Images of child sexual abuse or instructions in paedophilia
- Depictions of gratuitous or exploitative violence including sexual violence
- Depictions of actual or exploitative sexual practices including bestiality or incest
- Detailed instruction in or promotion of crime or violence including the use of illicit drugs or terrorist acts

**YES** Your app is **prohibited** by law and **must not** be available for download in Australia. Distribution, promotion or possession of this kind of app is a criminal offence https://www.acorn.gov.au/learn-about-cybercrime/prohibited-offensive-and-illegal-content Offensive content in your promotional materials is also prohibited.

**NO** Check your social media channels regularly. If offensive material is posted by users in relation to your app, you **must** remove this promptly. Go to Question 3.2.

**3.2 Your app must have an appropriate age-classification https://www.legislation.gov.au/Details/F2012L02541 Check your app for mature themes and language, violence, sex, drug use and nudity and check the classifications here http://www.classification.gov.au/Pages/Home.aspx Is your app classified, or likely to be classified, as R18+ or MA15+?**

**YES** You **must** ensure the app store implements access restrictions https://www.legislation.gov.au/Details/F2014L01757 to prevent under-age viewing. This also applies to how your app is promoted. Go to Advertising.

**NO** Go to Advertising.

Copyright © The University of Sydney, 2017

# Advertising

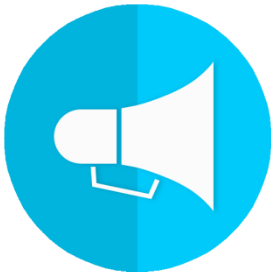

Australian Consumer Law covers how an app is advertised. Promotional materials:

- That are deceptive or misleading are **prohibited**
- **Should not** be offensive and should be age-appropriate
- **Should** provide information about financial costs associated with buying and using the app
- For medical devices have further restrictions

## WHAT ARE PROMOTIONAL MATERIALS?

Promotional materials are any information provided about an app to the public including: app store description; app store category; and information about the app on the developer's website, media releases, multimedia or social media channels. This **includes** testimonials, quotes and user reviews that have been copied by the app developer into promotional material but **excludes** user reviews posted directly onto an app store, and information written and disseminated by a third party. External postings about the app on an app developer's social media channels are also **excluded**, although these **should** be regularly monitored by the app developer and corrected if misleading. They **must** be removed if offensive.

**41. Do the app's promotional materials accurately reflect what the app provides?**

**YES** Health app developers in the United States have recently been charged over deceptive advertising claims https://www.ftc.gov/news-events/press-releases/2016/01/lumosity-pay-2-million-settle-ftc-deceptive-advertising-charges Go to Question 4.2.

**NO** You **must** edit all of your app's promotional materials to comply with the law. Here's how https://www.accc.gov.au/publications/advertising-selling Health app developers in the United States have recently been charged over deceptive advertising claims https://www.ftc.gov/news-events/press-releases/2016/01/lumosity-pay-2-million-settle-ftc-deceptive-advertising-charges

Copyright © The University of Sydney, 2017

**4.2 Is the promotional material likely to be seen by an audience that includes children?**

**YES** You **should** adhere to the industry standards http://aana.com.au/content/uploads/2014/05/AANA-Code-For-Marketing-Advertising-Communications-To-Children.pdf for advertising to children. If this is an app for adults, it should not be promoted in places where children will see it.

**NO** You **should** adhere to the industry standards http://aana.com.au/content/uploads/2014/05/AANA-Code-For-Marketing-Advertising-Communications-To-Children.pdf for advertising.

**4.3 Are there any up-front or in-app charges associated with downloading or using the app?**

**YES** You **must** be clear and transparent in your advertising about the cost of the app, including whether in-app purchases are required for full functionality.

**NO** Go to Question 4.4.

**4.4 Does downloading or usage of the app require extraordinary amounts of data?**

**YES** You **should** provide information upfront about data usage.

**NO** Go to Question 4.5.

**4.5 Does your app and related material act as a proxy advertisement for a health practitioner who is subject to regulation under the Australian Health Practitioner Regulation Agency (AHPRA)?**

**YES** You **must** adhere to the guidelines http://www.medicalboard.gov.au/Codes-Guidelines-Policies/Guidelines-for-advertising-regulated-health-services.aspx for advertising regulated health services.

**NO** Go to Financial.

Copyright © The University of Sydney, 2017

# Financial

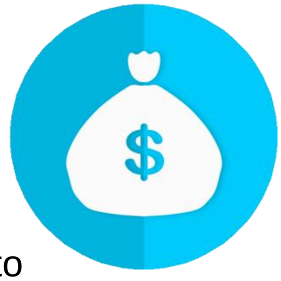

Some developers choose to make their app available to consumers for free, others for a price. Some apps also include in-app purchases, which allow consumers to upgrade or access extra content or buy subscriptions. You **should** provide information about in-app purchases in promotional materials and in the app. This information should be easily accessible and readily understood. If your app is targeted at potentially vulnerable users such as children or people living with mental illness, you **should not** repeatedly offer users in-app purchases.

**5.1 Does your app contain in-app purchases?**

**YES** You **should** provide information about in-app purchases in promotional materials and in the app. You **must** indicate whether in-app purchases are required for full functionality.

**NO** Go to Medical Devices.

**5.2 Do you sell your app directly to consumers (e.g. via your own website)?**

**YES** You **should** have an obvious and accessible process http://asic.gov.au/for-consumers/codes-of-conduct/epayments-code for refunding consumers the costs of downloading or using the app if it fails to meet consumer guarantees.

**NO** The app store where you sell your app **should** have an obvious and accessible process for refunding consumers the costs of downloading or using the app if it fails to meet consumer guarantees.

Copyright © The University of Sydney, 2017

# Medical Device

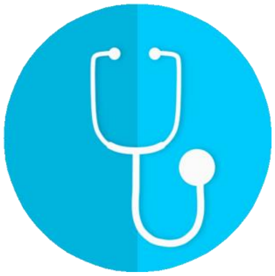

Some apps fit the legal definition of a “medical device.” Whether apps are covered by medical device laws depends on how likely use of the app will result in consumer harm. See whether your app is a “medical device” and what this means for you.

## IS MY APP A MEDICAL DEVICE?

User-generated data is any information entered into the app that comes from the user. This includes numbers or text entered directly by the user, active measuring or sampling of biological information, and passive entries from wearables. Apps may rely on user-generated data to generate tailored messages to users. Messages could be generated by algorithms, calculators, coaches or other means. If an app delivers health messages, it may be classified as a medical device. Example health messages include:

- Diagnosis: e.g. The user has…
- Prognosis: e.g. The user is at risk of …
- Monitoring: e.g. The user’s disease is getting better / worse, or is stable / unstable
- Advisory, including specific advice on how to alleviate or prevent a specific disease or modify a physiological process (“treatment” or “prevention”): e.g. The user should pursue a particular behaviour or use a product or service in a particular way (e.g. specifying dose or timing)

An app is unlikely to be classified as a medical device, if the app only ever:

- Indicates the risk that a population group has of developing a disease
- Provides general advice about a “healthy lifestyle” (such as limiting smoking and alcohol use, getting sufficient exercise)
- Provides links to support groups Gives generic advice to “seek help”
- Provides education about disease, anatomy or physiology Reminds users to take medications
- Monitors general health, fitness, wellbeing or the menstrual cycle (except if it investigates a specific physiological process)
- Stores user-generated data for later review by a health professional

Remember apps are defined as health-related more broadly when it comes to privacy than medical device law. Go to Privacy.

Copyright © The University of Sydney, 2017

**6.1 Is the focus of the app ANY of the following?**

- A specific disease, injury or disability? This DOES include medical diagnoses and conditions (e.g. depression, eating disorder). It does NOT include symptoms or conditions that are not classified as a medical disease (e.g. stress, trouble concentrating, difficulty sleeping)
- An anatomical or physiological process? This DOES include things like the sleep cycle. It does NOT include general well-being
- Control of conception

**YES** Go to Question 6.2.

**NO** It is unlikely that your app is a medical device. Go to Professionalism.

**6.2 Does the app claim that the output from the device can prevent or treat a specific disease, injury or disability or directly influence an anatomical or physiological process?**

**YES** Your app **may** be classified as a **medical device**, sometimes called "medical device software", a "mobile medical app" or "SAMD: software as a medical device." Your app will be subject to the Therapeutic Goods Administration (TGA) Medical Device regulation https://www.tga.gov.au/regulation-medical-software-and-mobile-medical-apps For more information, go here: https://www.tga.gov.au/publication/australian-regulatory-guidelines-medical-devices-argmd Go to Question 6.3.

**NO** Go to question 6.3

**6.3 Does the app collect user-generated data?**

**YES** Go to question 6.4

**NO** It is unlikely your app is a medical device. Go to Professionalism.

**6.4 Does the app deliver individualised health messages on the basis of user-generated data?**

**YES** Your app **may** be classified as a **medical device**, sometimes called "medical device software", a "mobile medical app" or "SAMD: software as a medical device." Your app will be subject to the Therapeutic Goods Administration (TGA) Medical Device regulation. For more information, go here: https://www.tga.gov.au/publication/australian-regulatory-guidelines-medical-devices-argmd Go to Question 6.5.

**NO** It is unlikely your app is a medical device. Go to Professionalism.

Copyright © The University of Sydney, 2017

### 6.5 Does the app allow direct diagnosis or monitor a vital physiological process?

**YES** Your app is likely a Class IIa (**low-medium risk**), Class IIb (**medium-high risk**) or Class III (**high risk**) medical device. All of these apps **must** be assessed by the Therapeutic Goods Administration https://www.tga.gov.au/publication/australian-regulatory-guidelines-medical-devices-argmd or must hold an equivalent certificate from a European Notified Body.

**NO** Your app is likely a Class I (**low risk**) medical device. You must conform to the Therapeutic Goods Administration's Essential Principles https://www.tga.gov.au/form/essential-principles-checklist-medical-devices for safety and performance but unless your app has a direct measuring function (e.g. wearables) your app does not require external assessment by the TGA. You must be able to provide evidence of conformity to the TGA upon request. Apps with a measuring function must undergo external assessment.

## NEXT STEPS

Your app is considered a medical device. Here's what to do next:

- Apply to the TGA to enter the app into the Australian Register of Therapeutic Goods: https://www.tga.gov.au/australian-register-therapeutic-goods
- Conform to the "Essential Principles" https://www.tga.gov.au/form/essential-principles-checklist-medical-devices for safety and performance (this includes designing the app to minimise the likely harm to the user).
- Obtain certification of conformity https://www.tga.gov.au/form/application-conformity-assessment-certificates-medical-devices to the Principles after assessment from the TGA or similar body https://www.tga.gov.au/conformity-assessment-requirements-australian-medical-device-manufacturers-streamlining-requirements (for Classes II, III or IV that are non-measuring devices) or via self-assessment (Class I and non-measuring devices).
- Notify the TGA https://www.tga.gov.au/conformity-assessment-requirements-australian-medical-device-manufacturers-streamlining-requirements if any adverse events are reported from the use of the app.
- Comply with the Therapeutic Goods Advertising Code https://www.tga.gov.au/publication/therapeutic-goods-advertising-code which restricts how you advertise the app.

For more information, you should contact the Therapeutic Goods Administration https://www.tga.gov.au

Copyright © The University of Sydney, 2017

# Professionalism

Health apps are an emerging and increasingly competitive market. There are standards of professionalism that set some health apps apart. Here's a checklist to see if your app can compete:

- ☐ I have identified myself as the developer and provided contact information in the app, in store and on promotional materials.

- ☐ I have identified the authors of the app content by:
  - Disclosing authorship, and providing author credentials
  - Citing all sources
  - Attributing all intellectual property

- ☐ I have disclosed all funding sources for the app, including commercial partners:
  - In the promotional materials
  - In the app itself

- ☐ I have disclosed (https://www.adma.com.au/compliance/code-of-practice) my business model (for example, up-front pricing, in-app purchases, subscription model, selling of personal information to third parties or commercial data collation) so consumers understand how they are paying for the service.

- ☐ I have provided scientific evidence to support the claims about what the app can do.
  - If I'm making a health claim, I have provided clinical evidence https://www.tga.gov.au/consultation/consultation-draft-clinical-evidence-guidelines-medical-devices#what-will-happen

- ☐ I have provided an easily accessible and understandable privacy policy https://www.oaic.gov.au/agencies-and-organisations/guides/guide-for-mobile-app-developers

- ☐ I have obtained consumers' fully informed consent https://www.adma.com.au/compliance/code-of-practice for collecting their data.

- ☐ I have carefully selected third party partners http://digitaladvertisingalliance.org/principles so that I only work with partners that are transparent and accountable about how they collect, store and share user data.

- ☐ I have designed my apps to be usable https://www.w3.org/WAI/mobile/ by all consumers including people with specific user needs such as those people with vision, hearing or dexterity impairments.

Copyright © The University of Sydney, 2017

# What are the Laws and Standards?

## Privacy

Commonwealth Privacy Act Australian Privacy Principles https://www.oaic.gov.au/privacy-law/privacy-act/australian-privacy-principles

Office of the Australian Information Commissioner Mobile Privacy: A Better Practice Guide for Mobile App Developers https://www.oaic.gov.au/agencies-and-organisations/guides/guide-for-mobile-app-developers

## Security

OWASP Mobile Security Project Top Ten Mobile Controls
https://www.owasp.org/index.php/Projects/OWASP_Mobile_Security_Project_-_Top_Ten_Mobile_Controls

Association for Data-driven Marketing & Advertising (ADMA) Code of Practice
https://www.adma.com.au/compliance/code-of-practice

Office of the Australian Information Commissioner (OAIC) Guide to Securing Personal Information
https://www.oaic.gov.au/agencies-and-organisations/guides/guide-to-securing-personal-information

## Content

Department of Communication and the Arts National Classification Code
http://www.classification.gov.au/Pages/Home.aspx

Australian Government Guidelines for the Classification of Films 2012
https://www.legislation.gov.au/Details/F2012L02541

## Advertising

Australian Competition & Consumer Commission (ACCC) Advertising & Selling
https://www.accc.gov.au/publications/advertising-selling

Australian Association of National Advertisers' Community Standards for Advertising to Children
http://aana.com.au/content/uploads/2014/05/AANA-Code-For-Marketing-Advertising-Communications-To-Children.pdf

Australian Association of National Advertisers' Code of Ethics
http://aana.com.au/content/uploads/2014/05/AANA-Practice-Note-Code-of-Ethics.pdf

Copyright © The University of Sydney, 2017

Association for Data-driven Marketing & Advertising (ADMA) Code of Practice https://www.adma.com.au/compliance/code-of-practice

## Financial

Australian Securities & Investments Commission ePayments Code http://asic.gov.au/for-consumers/codes-of-conduct/epayments-code/

Australian Communications and Media Authority Mobile apps: Occasional paper 1 http://www.acma.gov.au/theACMA/emerging-issues-in-media-and-communications-occasional-papers-1

## Medical Device

Therapeutic Goods Administration (TGA) Regulation of Medical Software and Mobile Medical 'Apps https://www.tga.gov.au/regulation-medical-software-and-mobile-medical-apps

Therapeutic Goods Administration (TGA) Australian Regulatory Guidelines for Medical Devices (under review) https://www.tga.gov.au/publication/australian-regulatory-guidelines-medical-devices-argmd

Therapeutic Goods Administration (TGA) Therapeutic Goods Advertising Code https://www.tga.gov.au/publication/therapeutic-goods-advertising-code

Therapeutic Goods Administration (TGA) Essential Principles Checklist (Medical Devices) https://www.tga.gov.au/form/essential-principles-checklist-medical-devices

Therapeutic Goods Administration (TGA) Conformity Assessment Certificates https://www.tga.gov.au/form/application-conformity-assessment-certificates-medical-devices

Therapeutic Goods Administration (TGA) Australian Register of Therapeutic Goods https://www.tga.gov.au/australian-register-therapeutic-goods

## Accessibility

World Wide Web Consortium (W3C) Mobile Accessibility Guidelines https://www.w3.org/WAI/mobile/

Copyright © The University of Sydney, 2017
